# Multiyear monitoring of bird communities in chlorpyrifos‐treated orchards in Spain and the United Kingdom: Spatial and temporal trends in species composition, abundance, and site fidelity

**DOI:** 10.1002/etc.4317

**Published:** 2019-01-23

**Authors:** Ralf Dittrich, Benedikt Giessing, María M. Benito, Anja Russ, Christian Wolf, Manousos Foudoulakis, Steve Norman

**Affiliations:** ^1^ tier3 solutions Leverkusen Germany; ^2^ Dow AgroSciences Thoriko Lavrion Greece; ^3^ Dow AgroSciences Abingdon United Kingdom; ^4^ RidgewayEco Harwell Innovation Centre Oxfordshire United Kingdom

**Keywords:** Organophosphorus insecticide, Chlorpyrifos, Wildlife toxicology, Risk assessment, Avian toxicity, Long‐term monitoring, Orchards

## Abstract

The relationship between agricultural practices and the welfare of wild birds has gained increased attention over the last decades. To assess the potential effects of chlorpyrifos on the bird community, a multiyear, multisite monitoring program was carried out in treated cider orchards (in the United Kingdom) and treated citrus orchards (in Spain). Constant‐effort mist netting was used over several consecutive years in the United Kingdom (2012–2014) and Spain (2010–2012). The general structure of the bird community and the presence of breeding species were analyzed. Twelve and 11 bird species (out of 81 and 45 trapped) in Spain and the United Kingdom, respectively, exceeded the 2% dominance value. For a selection of 6 species in citrus and 4 in cider orchards, *N*‐mixture models were fitted to the number of trapped birds. The abundance of most species was strongly and significantly affected by seasonality. No species showed any indication of reduction in population size over the years. The results of this extensive field program support the indications that chlorpyrifos spray applications present a low risk to the bird community over the years. *Environ Toxicol Chem* 2019;38:616–629. © 2018 The Authors. *Environmental Toxicology and Chemistry* Published by Wiley Periodicals, Inc. on behalf of SETAC.

## INTRODUCTION

The assessment of acute and short‐term risks posed by a specific pesticide to wild birds can be based on different methods, such as telemetric monitoring on a wide scale in a multiple‐crop and multiple‐country approach as described by Wolf et al. (2010). However, to assess the long‐term risks of bird communities exposed to a specific pesticide, new approaches and appropriate methods for field studies are needed. The aim of the monitoring program we describe was to assess the potential long‐term risk posed by chlorpyrifos to populations of wild birds in a scientifically robust and environmentally relevant way using a holistic approach. This consisted of collecting data to describe the bird community, by applying methods like constant‐effort trapping, nest monitoring, telemetry, and bird survey, combined with data about arthropod presence over time and habitats surrounding the study sites. This comprehensive dataset can be combined with results of laboratory dosing studies, modeling exercises, controlled field studies, and incident reports. This weight of evidence approach is generally thought to provide a highly realistic assessment of the risk posed by a pesticide.

### General background information on avian monitoring in orchards

To the best of our knowledge, the monitoring program we describe is the first to thoroughly characterize the local bird populations in orchards in terms of aspects such as composition, temporal dynamics of abundance, and site fidelity, at multiple sites and over multiple years. Previous studies on bird communities in orchards have relied on visual observations (Genghini et al. 2006; Wiacek and Polak 2008; Bouvier et al. 2011). This is a straightforward method, but does not yield the detailed information that systematic bird trapping (mist‐netting) provides. The aim of using the same habitat type within 10 sites in a single geographical area was to uncover the relevant sources of variation in terms of surrounding habitats and different farming practices. Furthermore, the monitoring program aimed to investigate potential effects of chlorpyrifos on bird communities both in citrus orchards in Spain (Valencia) and in pome fruit orchards in the United Kingdom (Herefordshire).

### Selection of study areas

The Valencia area is characterized by intensive citrus orchard farming. The landscape consists of a coastal plain against a backdrop of steeply sloped hills. The orchards on the plain tend to be abutted by parcels of agricultural land (mostly citrus, but also other crops like vegetables) that often include houses and gardens. The surrounding habitats of the citrus orchards on the hillsides are generally composed of macchia vegetation, set‐asides, riparian vegetation, and small coniferous forest patches. Almost all commercial citrus orchards in the Valencia area are sprayed with chlorpyrifos to control a major economic pest, the California red scale insect (*Aonidiella aurantii*). The usual practice is to make a single chlorpyrifos application every year in late May or early June. Depending on pest‐pressure, chlorpyrifos is applied a second time later in the season. In this monitoring program, orchards were sprayed at 2400 g a.i./ha, with 1 or 2 applications.

The Herefordshire area is characterized by a mosaic of orchards, meadows, and arable fields with surrounding habitats such as hedges, riparian vegetation, and small forest patches in a flat landscape. It contains more pome fruit orchards (covering ∼5500 ha) than any other county in the United Kingdom. The great majority of these Herefordshire orchards grow apples for cider production, and thus are hereafter termed “cider orchards.” Almost all the commercial (excluding organic) cider orchards in the Herefordshire area were sprayed with chlorpyrifos before blossom time to control a major economic pest, the apple blossom weevil (*Anthonomus pomorum*). Depending on pest‐pressure (e.g., from apple sawfly), a second application was sometimes made after blossom. For the present monitoring program, the first spraying was done at 480 g a.i./ha, and the second at 960 g a.i./ha.

In both study areas, the decision as to whether a second chlorpyrifos application was needed depended on local agronomic need and was solely determined by the farmers. The authors and the study sponsors played no role in agronomic practice decisions. Thus these orchards were ideal for conducting a monitoring program with the aim of investigating potential effects of chlorpyrifos on the avifauna.

## MATERIALS AND METHODS

All bird species found in the study orchards and their close surroundings were included in the study. The program was undertaken over several consecutive years in the United Kingdom (2012–2014) and Spain (2010–2012), and was performed using essentially the same methodology and study design in both countries, making the results fully comparable. The citrus and cider orchards were large enough (3.5–10 ha) for a significant number of birds to breed and forage. The application date(s) and applied amount of chlorpyrifos were recorded in all cases.

### Study area, period, and application in Spain

**Figure 1:**
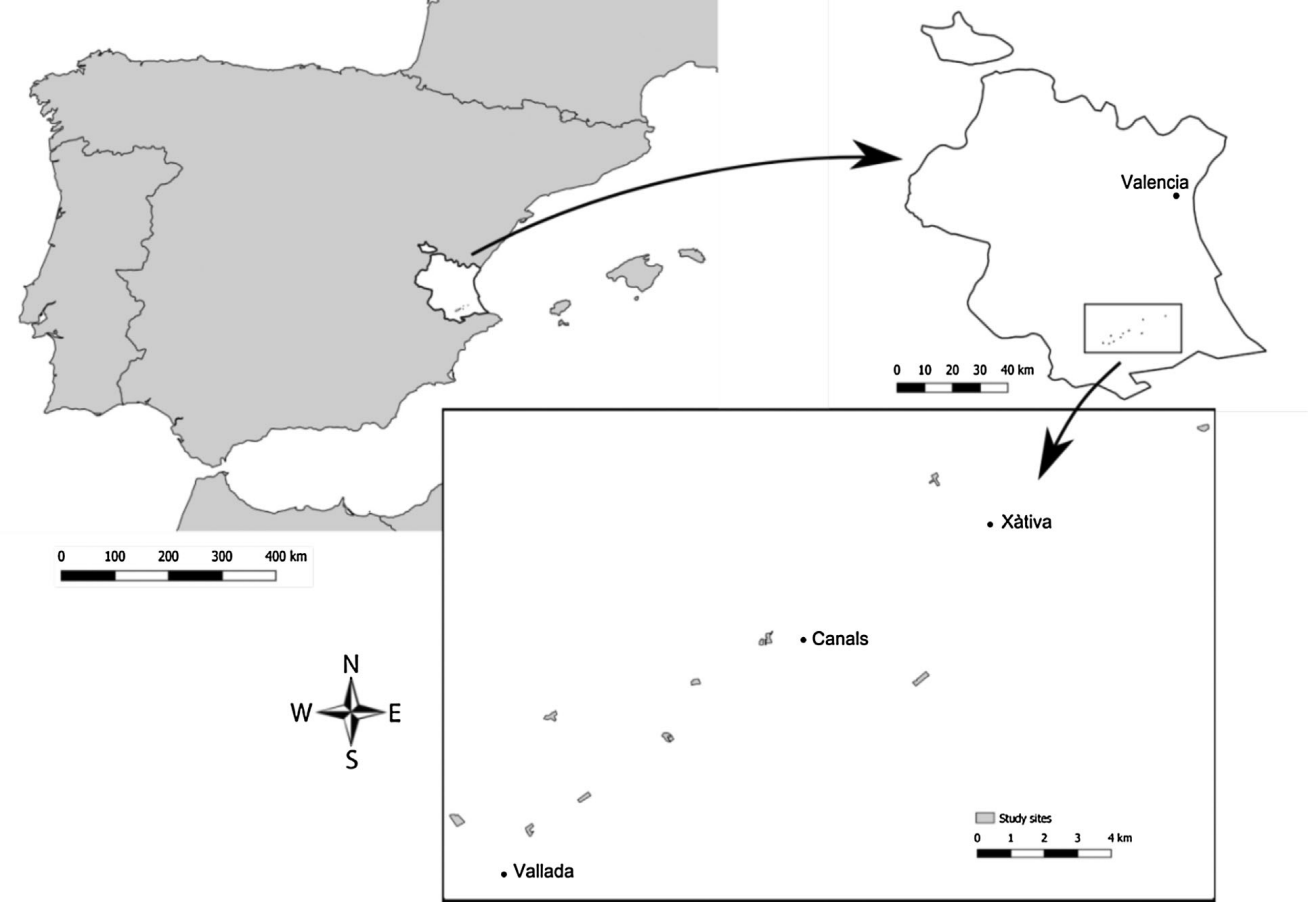
Location of the study area in Spain in the province of Valencia, and the citrus orchards studied.

Fieldwork was conducted in citrus orchards around Xàtiva, 50 km south of Valencia. The area is approximately 120 m above sea level, and has a steppe climate, with mild winters and hot, dry summers (Peel et al. 2007). Ten commercially used and typical citrus orchards, all drip‐irrigated, were chosen as study sites (Figure 1 and Supplemental Data, Table S1). The maximum height of the trees was approximately 2.5 to 6 m. The age of the trees ranged from approximately 8 to 25 yr. Data collection took place from July to August 2010, April to August 2011, and March to July 2012. The chlorpyrifos formulation used in the present study is registered in Spain for 2 applications in citrus orchards at 2400 g a.i./ha. According to good agricultural practice, the first application is made between the end of May and the beginning of June, and a second application, if necessary, between August and September.

### Study area, period, and application in the United Kingdom

**Figure 2:**
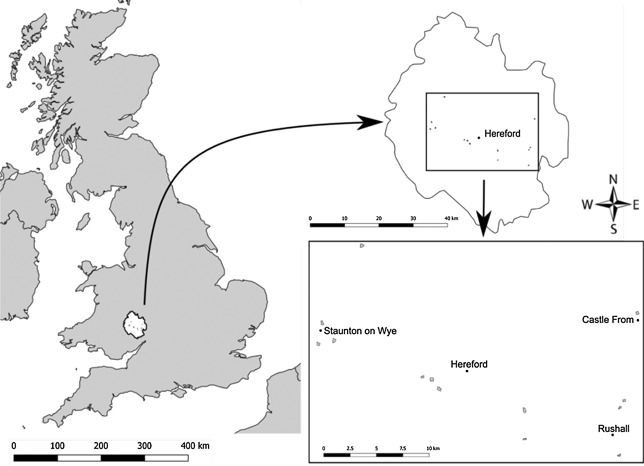
Location of the study area in the United Kingdom in Herefordshire County, and the cider orchards studied.

In Herefordshire, cider orchards were investigated. The area is approximately 58 m above sea level and has an oceanic climate with warm summers and significant rainfall (Peel et al. 2007). Ten conventional and 3 organic cider orchards (the latter as untreated reference sites), were chosen as study sites (Figure 2 and Supplemental Data, Table S2). In the conventional orchards, the trees were approximately 5 to 7 m high and approximately 15 to 45 yr old. Data collection took place from April to July 2012, April to July 2013, and from the end of March to July 2014. The chlorpyrifos formulation used in the present study was registered in the United Kingdom for application in apple orchards at 480 g a.i./ha (before flowering) and 960 g a.i./ha (after flowering). As in Spain, farmers were asked to make applications of chlorpyrifos in the context of their common agronomic practice. The preflowering application was made to all 10 study orchards in 2012, 2013, and 2014, in general between the end of March and mid‐May. In most of the orchards, chlorpyrifos was applied a second time post flowering, usually between mid‐May and the beginning of June.

### Bird trapping (mist netting)

The species composition, number of individuals, and age of birds was assessed by bird trapping. Moreover, by trapping the birds it was possible to investigate whether individuals marked (i.e., leg‐ringed) in previous years had returned to the same sites, illustrating survival and site fidelity. Mist netting was not done at the 3 reference sites (organic orchards in the United Kingdom) because the canopy of the trees began too high off the ground. Netlines of 4 consecutive mist nets (total 36 m, 16‐mm mesh width, height 2.5 m), were used. Due to general lower bird abundance, the number and total length of netlines were higher in the United Kingdom (10 netlines, 360 m) than in Spain (288 m). The location of the mist nets was the same throughout the study: it was determined at the first trapping by fixed holes for the net poles in the ground.

Trapping was done approximately every 12th day in each study orchard in Spain (total 1594 h of trapping effort in 2010–2012). In the United Kingdom the trapping schedule was more flexible, to accommodate farmers’ crop husbandry; in total there were 7 trapping sessions in 2012 and 6 sessions in 2013 and 2014 (880 h total). The sessions were mostly conducted during the early morning, the main active period of the birds (Ralph and Dunn 2004). Additional trapping sessions in the evening were conducted at 3 of the United Kingdom sites (sites 1, 2, and 7) and in all Spanish orchards in July and August to better cover the times of bird activity during the hot summer period.

### Analysis

#### Species composition

Trapping data were used to calculate species‐specific parameters such as frequency of occurrence (percentage of study orchards or trapping sessions in which the species occurred) and dominance (percentage of individuals with respect to all birds, including first retraps in a year).

The bird communities in the study orchards were characterized by different parameters and indices: number of species (species richness), total number of birds trapped (abundance), the Shannon–Wiener diversity index, the Simpson evenness index (another measure of diversity that is less sensitive to rare species than the Shannon–Wiener index), and the Pilou evenness index, which indicates the variation in abundances between different taxa within the community. To test for between‐year changes in the community structure (year‐wise analysis) these indices were obtained for each orchard and year. Furthermore, except for the Pilou evenness index, these indices were also calculated per site and trapping session to test for short‐term effects of the exposure to chlorpyrifos (session‐wise analysis).

Generalized linear mixed models (GLMMs) were fitted to the community indices (species richness, abundance, Shannon–Wiener index, and Simpson and Pilou evenness indices) to assess whether the application of chlorpyrifos was correlated with changes in the community structure. Year‐wise species richness and abundance were modeled as GLMMs with a Poisson error distribution, and the Shannon–Wiener diversity index as a linear mixed model, whereas beta regression GLMMs were fitted to the Simpson and Pilou evenness indices due to their distribution between 0 and 1. The model included the factor “country” to distinguish between apple and citrus orchards, the “year” as a linear and quadratic term, and the “orchard” as a random intercept to account for the nonindependence of repeated measurements/orchard. The linear and quadratic terms of year were centered and scaled to allow for comparison between parameter estimates (Schielzeth 2010).

The session‐wise community indices were modeled as just described for the year‐wise indices: species richness and abundance as a Poisson GLMM, and the Simpson evenness index as a beta regression GLMM. A zero‐inflated GLMM was fitted to the Shannon–Wiener diversity index to account for excess zeros in the data due to trapping sessions in which only one bird was caught. A factor “exposure” was defined indicating whether the capture date was after the application of chlorpyrifos. For the data obtained in cider orchards, the application of chlorpyrifos was spread over almost the entire season. Therefore, the factor exposure was defined to indicate whether the capture date was within 10 d after application of chlorpyrifos to account for possible short‐term effects. In Spanish citrus orchards, the application of chlorpyrifos was restricted to a short time‐span between the end of May and the beginning of June. Here, the factor exposure was defined to indicate whether the capture date lay after chlorpyrifos application in the orchard. The model included the “capture date” as linear (“day of year”), quadratic (“day of year^2^”), and cubic (“day of year^3^”) terms to allow nonlinear relationships between community indices and season. Furthermore, we included the year, country, and exposure as cofactors, the orchards as random intercept, and all 2‐way interactions between the cofactors and the capture date. The linear and higher order terms of capture date were centered and scaled to allow for comparison between parameter estimates (Schielzeth 2010). Poisson GLMMs were checked for overdispersion, and, when necessary, an observation‐level random effect was included in the model. Validation plots (quantile–quantile [QQ] plots, histogram of residuals, and residuals vs fitted values) were assessed to verify the appropriateness of the chosen model (Zuur et al. 2009).

Species were assigned to a feeding guild following Bouvier et al. (2011), and diet information was taken from Cramp et al. (1977–1994). To allow a valid comparison with the study period, the prevalent diet during the breeding period was selected. Finches were categorized as granivorous because they feed mainly on seeds all year round, whereas all thrushes (due to earthworms and snails in their diet) and corvids were classified as omnivores.

Because the application of chlorpyrifos might affect insectivorous bird species the most (because of a lack of prey), the calculations of year‐wise and session‐wise diversity indices and subsequent analyses were repeated with insectivorous species only.

#### Measure of site fidelity of individuals

As a measure of site fidelity, the percentage of adult individuals trapped at least twice on different days in the same orchard within a year (“within‐season retraps”) or again in the next year (“between‐season retraps”) was calculated. Thus the number and percentage of individuals/species associated with the study orchards was identified. The age of individuals when trapped for the first time was also taken into account.

#### Seasonality and year‐to‐year comparison of abundance of common bird species

For the most common bird species, the number of individuals trapped was modeled to investigate whether exposure to chlorpyrifos had any short‐ or long‐term effects on population size. In the citrus orchards, the 5 most abundant species, with a dominance of approximately 10% or greater, were the serin (*Serinus serinus*), Sardinian warbler (*Sylvia melanocephala*), house sparrow (*Passer domesticus*), blackbird (*Turdus merula*), and greenfinch (*Carduelis chloris*). In cider orchards, the 4 most abundant species were the blue tit (*Cyanistes caeruleus*), great tit (*Parus major*), robin (*Erithacus rubecula*), and blackbird. For these species plus the great tit in citrus orchards (representing a common insectivorous breeding species with 3.8% dominance), enough data were available to model the population size in the study orchards with hierarchical models (Kéry and Royle 2016) to identify potential effects of chlorpyrifos application on the population size and detectability. Because only adult bird data were included in the analysis and the trapping session was mostly restricted to the breeding season, we assumed closed populations/orchard and used the pcount function to fit *N*‐mixture models (Royle 2004). These models allow for imperfect detection and estimate the population size *N* and a detectability parameter *φ*. The population size *N*, which is assumed to be constant over the season, was modeled either as a constant or was allowed to differ per year, whereas *φ* was modeled to vary by capture date, year, exposure, and all 2‐way interactions as well as the 3‐way interaction among capture date, exposure, and year. The capture date was included as a linear (day of year), quadratic (day of year^2^), and cubic (day of year^3^) term. Capture date and year were centered and scaled prior to including these variables into the model. The best model among this set of models was chosen by the lowest Akaike information criterion value (Burnham and Anderson 2002). The goodness of fit of the best *N*‐mixture model/per species was assessed by residual plots, QQ plots, and an estimate of overdispersion (ĉ), as suggested by Knape et al. (2018).

#### Statistical analysis

Statistical data analyses were conducted in R (Ver 3.4.4; R Development Core Team 2017) using the additional packages vegan (Ver 2.3‐4; Oksanen et al. 2016), lme4 (Ver 1.1‐12; Bates et al. 2015), glmmTMB (Ver 0.2.2.0; Brooks et al. 2017), and unmarked (Ver 0.11‐0; Fiske and Chandler 2011).

Model predictions were plotted with 95% confidence intervals, which might underestimate the uncertainty around the estimates but provides a reasonable approximation (Bates et al. 2015). Significant *p* values were established at the 5% level for all analyses, and are marked with an asterisk in the tables.

## RESULTS

### Species composition

In total, 13 544 individual birds of 81 species were trapped in the 10 citrus orchards in Spain. Whereas 39.5% of the species (*n* = 32) were infrequent (i.e., trapped in 4 or less orchards), almost an equivalent number (*n* = 31, 38.3%) was common (i.e., trapped in at least 8 orchards). The abundance and species richness varied greatly between the orchards, ranging between 522 and 2453 individuals (mean ± standard deviation, 1354 ± 511) and 35 to 54 species (45 ± 6) trapped/site. In the United Kingdom, where in total 3230 individuals of 45 species were trapped, the trapping success also differed greatly among orchards, from 114 to 440 individuals (323 ± 110) and 15 to 28 species (22 ± 5). More species were infrequent than in Spain (*n* = 21, 46.7%), but a similar proportion of species was common (*n* = 16, 35.5%).

Twelve and 11 species in Spain and the United Kingdom, respectively, made up for 83 and 87% of all trapped birds, thus exceeding the 2% dominance value and being considered as dominant and relevant for the bird community in each country (Table 1).

**Table 1:** Dominant bird community in citrus and pome (cider) orchards in Spain and the United Kingdom

Country	Species	Dominance (% of all trapped birds)	Feeding guild	FO_trapping_ ^a^	FO_study orchards_ ^a^	Breeding inside orchard^b^
Spain						
	Serin	17.6	Granivorous	85.2	100.0	Common
	Sardinian warbler	11.3	Insectivorous	85.2	100.0	Rare^c^
	House sparrow	10.1	Insectivorous	52.8	100.0	No
	Blackbird	10.1	Omnivorous	83.5	100.0	Common
	Greenfinch	9.5	Granivorous	54.0	100.0	Common
	Goldfinch	6.0	Granivorous	45.5	100.0	Common
	Great tit	3.8	Insectivorous	58.5	100.0	Rare
	Blackcap	3.7	Insectivorous^c^	15.9	100.0	No
	Barn swallow	3.4	Insectivorous	33.5	100.0	No
	Linnet	2.8	Granivorous	13.1	70.0	Rare
	Tree sparrow	2.6	Insectivorous	26.7	90.0	No
	Nightingale	2.2	Insectivorous	28.4	100.0	No
	Total	83.3	**—**			**—**
United Kingdom						
	Blue tit	19.4	Insectivorous	77.1	100.0	Common
	Great tit	17.2	Insectivorous	79.8	100.0	Common
	Robin	16.5	Insectivorous	77.6	100.0	Rare^d^
	Blackbird	14.6	Omnivorous	72.2	100.0	Rare^d^
	Chaffinch	5.4	Granivorous	49.8	100.0	No
	Common redstart	2.9	Insectivorous	23.8	100.0	Rare
	Long‐tailed tit	2.7	Insectivorous	13.0	90.0	No
	Song thrush	2.4	Omnivorous	26.0	100.0	No
	Blackcap	2.2	Insectivorous	20.6	90.0	No
	Chiffchaff	2.1	Insectivorous	21.5	80.0	No
	Mistle thrush	2.0	Omnivorous	18.8	90.0	No
	Total	87.4	—			—

^a^ Frequency of occurrence = percentage of study orchards or trapping sessions in which the species occurred.

^b^ Common = in more than 3 orchards; rare = in up to 3 orchards.

^c^ Found mainly breeding in dense vegetation close by.

^d^ Trapped mostly on migration, then also frugivorous and nectar feeding.

Insectivorous birds were by far the most abundant species in both orchard types, comprising 69% of the individuals trapped in the United Kingdom and 52% of those trapped in Spain. Omnivorous birds were relatively abundant in the United Kingdom (22%) and were occasional in Spain (12%). In contrast, the presence of granivorous birds was low in the United Kingdom (9%), whereas in Spain they contributed more than one‐third of the trapped birds (36%).

Irrespective of absolute trapping numbers, the trapping frequency of each species provides information about the regularity of the use of the treated orchards. Among the 5 most abundant species in Spain, the house sparrow and the greenfinch were only present in 53 and 54% of trapping events, respectively. The results also showed local effects; for instance, the linnet (*Linaria cannabina*) belongs to the dominant community but was only trapped in greater numbers in one orchard. As a winter guest or during migration, the blackcap (*Sylvia atricapilla*) was mainly trapped in March/April. In cider orchards, the trapping frequency was generally lower, but all 4 common species were present in approximately 72% or more trapping events. Other species had considerably lower frequencies (Table 1).

None of the species frequently trapped is of high priority for conservation according to Annex I of the European Union 2009/147 directive (European Parliament 2009). However, in the citrus orchards the species richness was quite high, and 2 species are included on the Spanish Red list: the Iberian grey shrike (*Lanius meridionalis* [BirdLife 2017a]; considered vulnerable) and the woodchat shrike (*Lanius senator* [BirdLife 2017b]; considered near threatened). The bird community in the cider orchards was characterized by low species richness, but 2 of the frequent species are of high conservation concern, the song thrush (*Turdus philomelos*) and the mistle thrush (*Turdus viscivorus*).

The total bird diversity in the orchards over all study years was higher in citrus than in cider orchards (Shannon–Wiener diversity index = 2.83 and 2.44, Simpson evenness index = 0.91 and 0.86, respectively), but the bird community in cider orchards was more even (Pilou evenness index = 0.23; i.e., contained less rare or very abundant species) than in citrus orchards (Pilou evenness index = 0.21).

The species composition/year and orchard as indicated by the year‐wise community indices did not deteriorate over the years (Table 2). Instead, the species richness, abundance, and Shannon–Wiener diversity and Simpson evenness indices were relatively stable in the cider orchards between 2012 and 2014 and increased significantly in the citrus orchards from 2010 to 2012 probably due to the larger trapping efforts in 2011 and 2012. Only the Pilou evenness index decreased in citrus orchards between 2010 and 2012, indicating that the increase in species richness was due to the additional trapping of rare species. This overall pattern was also consistent when only insectivorous species were taken into account (Table 2).

**Table 2:** Effect estimates from (generalized) linear mixed model analysis for indices of bird community structure/year and orchard^a^

	Total				Insectivores			
	Estimate	SE	Z	*p* value	Estimate	SE	Z	*p* value
Species richness								
Intercept	3.253	0.074	43.84	<0.001*	2.869	0.078	36.76	<0.001*
Country (UK)	–0.740	0.121	–6.112	<0.001*	–0.917	0.135	–6.781	<0.001*
Year	0.735	0.144	5.100	<0.001*	0.940	0.183	5.139	<0.001*
Year^2^	–0.602	0.150	–4.018	<0.001*	–0.781	0.194	–4.028	<0.001*
Abundance								
Intercept	5.748	0.137	42.079	<0.001*	5.027	0.129	38.92	<0.001*
Country (UK)	–1.452	0.202	–7.185	<0.001*	–1.201	0.194	–6.180	<0.001*
Year	1.023	0.156	6.577	<0.001*	1.495	0.179	8.364	<0.001*
Year^2^	–0.651	0.152	–4.276	<0.001*	–0.969	0.173	–5.606	<0.001*
Shannon–Wiener diversity index								
Intercept	2.455	0.074	33.06	<0.001*	2.112	0.088	23.86	<0.001*
Country (UK)	–0.522	0.111	–4.715	<0.001*	–0.683	0.134	–5.118	<0.001*
Year	0.583	0.098	5.940	<0.001*	0.639	0.130	4.930	<0.001*
Year^2^	–0.475	0.095	–5.006	<0.001*	–0.537	0.125	–4.286	<0.001*
Simpson evenness index								
Intercept	1.933	0.101	19.24	<0.001*	1.477	0.132	11.20	<0.001*
Country (UK)	–0.532	0.149	–3.574	<0.001*	–0.675	0.196	–3.440	0.001*
Year	0.583	0.141	4.124	<0.001*	0.662	0.194	3.412	0.001*
Year^2^	–0.448	0.13	–3.377	0.001*	–0.517	0.181	–2.861	0.004*
Pilou evenness index								
Intercept	–1.000	0.035	–28.38	<0.001*	–0.927	0.053	–17.60	<0.001*
Country (UK)	0.275	0.055	5.029	<0.001*	0.382	0.083	4.608	<0.001*
Year	–0.254	0.065	–3.896	<0.001*	–0.385	0.106	–3.620	<0.001*
Year^2^	0.213	0.062	3.429	0.001*	0.333	0.101	3.289	0.001*

^a^ The reference level for country is Spain (citrus orchards). Marked with an asterisk are significance levels < 0.05.

SE = standard error.

The session‐wise community indices showed a clear seasonal trend indicated by the significant correlation for all indices and the different day of year terms (Table 3 and Supplemental Data, Figure S1). In the citrus orchards, the species richness, abundance, and Shannon–Wiener diversity and Simpson evenness indices peaked in April and decreased slowly afterward, whereas in the cider orchards the peak was slightly delayed toward the beginning of May for species richness, abundance, and Shannon–Wiener diversity index (indicated by a significant interaction between country and day of year; Table 3 and Supplemental Data, Figure S1). The abundance of birds was the sole community index that showed a significant correlation with exposure to chlorpyrifos (Table 3), although, because this correlation was positive, it did not indicate an adverse effect of the application of chlorpyrifos on the abundance of trapped birds. The community structure at the study orchards was relatively stable over the years. There was no adverse long‐term effect; instead, the abundance of trapped birds was significantly greater in 2012 than in 2010, although this might be confounded by the late trapping in 2010 and higher trapping efforts in 2011 and 2012. The Shannon–Wiener diversity index significantly increased from 2011 to 2012 (Table 3 and Supplemental Data, Figure S1), indicating a larger diversity in 2012. However, this difference was not reflected when only insectivores were analyzed. The Shannon–Wiener diversity and the Simpson evenness indices showed a different seasonal pattern in 2012 compared with 2013 and 2014. Whereas these indices decreased in 2013 and 2014 during June and July, they remained relatively constant in 2012 (Supplemental Data, Figure S1). This pattern was even more pronounced in the analysis of the insectivorous species (Table 3 and Supplemental Data, Figure S1).

**Table 3:** Effect estimates from (generalized) linear mixed model analysis for indices of bird community structure/trapping session and orchard^a^

	Total (*n* = 920)				Insectivores (*n* = 801)			
	Estimate	SE	Z	*p* value	Estimate	SE	Z	*p* value
Species richness								
Intercept	1.574	0.137	11.50	<0.001*	1.160	0.157	7.390	<0.001*
Exposure	0.138	0.097	1.431	0.153	–0.094	0.140	–0.673	0.501
Day of year	3.329	0.723	4.606	<0.001*	–1.550	1.156	–1.341	0.180
Day of year²	–6.636	1.618	–4.103	<0.001*	3.242	2.529	1.282	0.200
Day of year³	2.891	0.903	3.203	0.001*	–1.569	1.383	–1.135	0.257
2010	–0.145	0.103	–1.409	0.159	0.178	0.164	1.089	0.276
2011	–0.057	0.052	–1.088	0.277	–0.115	0.078	–1.469	0.142
2013	–0.047	0.107	–0.434	0.664	0.036	0.138	0.261	0.794
2014	–0.201	0.117	–1.721	0.085	0.019	0.149	0.126	0.900
Country	–0.039	0.164	–0.236	0.814	0.094	0.170	0.554	0.579
Exposure × day of year	0.155	0.099	1.562	0.118	0.024	0.146	0.166	0.868
Day of year × 2010	–0.336	0.152	–2.213	0.027*	–0.320	0.213	–1.504	0.133
Day of year × 2011	0.051	0.051	0.993	0.321	–0.079	0.085	–0.930	0.352
Day of year × 2013	–0.280	0.113	–2.476	0.013*	–0.237	0.155	–1.528	0.126
Day of year × 2014	–0.307	0.114	–2.693	0.007*	–0.078	0.156	–0.497	0.619
Day of year × country	0.450	0.107	4.219	<0.001*	0.039	0.155	0.253	0.801
Abundance								
Intercept	1.913	0.186	10.260	<0.001*	1.792	0.253	7.091	<0.001*
Exposure	0.437	0.130	3.358	0.001*	–0.168	0.227	–0.739	0.460
Day of year	2.887	0.950	3.039	0.002*	–2.552	1.883	–1.355	0.175
Day of year²	–5.941	2.118	–2.805	0.005*	5.367	4.106	1.307	0.191
Day of year³	2.069	1.174	1.762	0.078	–2.476	2.236	–1.108	0.268
2010	–0.349	0.128	–2.733	0.006*	0.128	0.266	0.482	0.629
2011	–0.063	0.070	–0.896	0.370	–0.195	0.125	–1.563	0.118
2013	0.163	0.142	1.146	0.252	0.029	0.234	0.125	0.901
2014	–0.121	0.153	–0.792	0.428	0.018	0.250	0.070	0.944
Country	–0.059	0.227	–0.260	0.795	0.064	0.276	0.232	0.816
Exposure × day of year	0.473	0.128	3.696	<0.001*	–0.056	0.235	–0.237	0.813
Day of year × 2010	–0.215	0.188	–1.148	0.251	–0.399	0.341	–1.169	0.242
Day of year × 2011	0.171	0.072	2.392	0.017*	–0.163	0.136	–1.199	0.230
Day of year × 2013	–0.280	0.143	–1.960	0.050	–0.231	0.263	–0.881	0.378
Day of year × 2014	–0.345	0.143	–2.421	0.015*	–0.100	0.257	–0.390	0.697
Day of year × country	0.522	0.135	3.869	<0.001*	–0.040	0.251	–0.157	0.875
Shannon–Wiener diversity index								
Intercept	1.497	0.135	11.31	<0.001*	0.877	0.125	7.040	<0.001*
Exposure	0.036	0.096	0.378	0.705	0.009	0.094	0.096	0.923
Day of year	3.488	0.738	4.724	<0.001*	5.527	0.777	7.116	<0.001*
Day of year²	–6.999	1.615	–4.334	<0.001*	–11.716	1.697	–6.904	<0.001*
Day of year³	3.265	0.881	3.707	<0.001*	5.875	0.924	6.359	<0.001*
2010	–0.141	0.090	–1.574	0.116	–0.051	0.111	–0.464	0.643
2011	–0.124	0.050	–2.465	0.014*	–0.055	0.052	–1.066	0.286
2013	–0.100	0.103	–0.974	0.330	–0.169	0.098	–1.721	0.085
2014	–0.207	0.109	–1.906	0.057	–0.237	0.105	–2.251	0.024*
Country	–0.089	0.163	–0.545	0.586	0.131	0.148	0.991	0.378
Exposure × day of year	0.038	0.094	0.405	0.685	0.162	0.099	1.644	0.100
Day of year × 2010	–0.385	0.129	–2.995	0.003*	–0.263	0.142	–1.843	0.065
Day of year × 2011	–0.000	0.052	–0.007	0.994	0.055	0.056	0.977	0.329
Day of year × 2013	–0.293	0.104	–2.834	0.005*	–0.405	0.109	–3.704	<0.001*
Day of year × 2014	–0.287	0.102	–2.817	0.005*	–0.406	0.107	–3.976	<0.001*
Day of year × country	0.411	0.098	4.208	<0.001*	0.522	0.104	5.032	<0.001*
Simpson evenness index								
Intercept	0.648	0.288	2.246	0.025*	–0.300	0.277	–1.084	0.278
Exposure	0.054	0.231	0.234	0.815	–0.093	0.226	–0.412	0.680
Day of year	6.543	1.628	4.019	<0.001*	9.280	1.951	4.756	<0.001*
Day of year²	–12.57	3.580	–3.511	<0.001*	–19.177	4.258	–4.504	<0.001*
Day of year³	5.749	1.954	0.942	0.003*	9.242	2.321	3.981	<0.001*
2010	–0.083	0.198	–0.421	0.674	–0.13	0.275	–0.503	0.615
2011	–0.203	0.118	–1.722	0.085	–0.058	0.125	–0.467	0.641
2013	0.069	0.230	0.300	0.764	–0.229	0.234	–0.979	0.328
2014	–0.044	0.247	–0.178	0.859	–0.369	0.252	–1.465	0.143
Country	–0.257	0.329	–0.780	0.435	0.205	0.316	0.649	0.516
Exposure × day of year	0.102	0.221	0.465	0.644	0.350	0.237	1.481	0.139
Day of year × 2010	–0.991	0.290	–3.422	0.001*	–0.492	0.358	–1.384	0.166
Day of year × 2011	–0.097	0.127	–0.764	0.445	0.160	0.138	1.162	0.245
Day of year × 2013	0.207	0.237	–0.873	0.383	–0.763	0.263	–2.902	0.004*
Day of year × 2014	–0.182	0.232	–0.785	0.433	–0.772	0.256	–3.012	0.003*
Day of year × country	0.306	0.225	1.356	0.175	0.872	0.248	3.520	<0.001*

^a^ Reference levels are Spain (citrus orchards) for country, 2012 for year and “not exposed” for exposure.

* Significance levels < 0.05.

SE = standard error.

### Site fidelity of individuals

Considerable differences in site fidelity were found between species (Table 4). The percentage of within‐season retraps shows the minimum proportion of individuals of a given species that regularly used the same orchard within the same breeding season. It can be used as an indicator for the “intensity” with which a given species is connected to the treated orchard. In both countries, the great tit (∼40% of adult birds trapped at least twice in a year) was the most regular orchard user, followed by the Sardinian warbler (31.8%) in Spain and the blue tit (31.8%) and robin (36.6%) in the United Kingdom. Other species showed considerable differences between countries (e.g., the blackbird, with 30.2% of within‐season retraps in Spain and only 18.1% in the United Kingdom). Regular orchard users were less common among the serin in Spain (16.5%) and chaffinch in the United Kingdom (10.6%). Overall, 15.4 and 21.5% of adult birds of the common species were retrapped in the following year (between‐season retraps) in Spain and the United Kingdom, respectively. This is comparable to the overall within‐season retrapping rates of 17.6 and 28.3% in Spain and the United Kingdom, respectively. The ranking order for most species according to the within‐ and between‐season retraps was comparable (Table 4).

**Table 4:** Proportion of individuals retrapped within a season or among years in citrus orchards^a^

		Proportion of adult birds retrapped within season^b^		Proportion of adult birds retrapped in the following year^c^		Proportion of juvenile birds retrapped in the following year^d^	
Country	Species	%^d^	No.	%^e^	No.	%^e^	No.
Spain							
	Blackbird	30.2	197	31.1	141	8.6	34
	Serin	16.5	149	13.2	98	2.3	15
	Sardinian warbler	31.8	108	25.5	48	4.3	25
	Great tit	44.5	93	32.3	47	17.1	30
	Blackcap	17.0	71	30.0	3	15.2	5
	Greenfinch	9.6	51	10.5	49	3.3	9
	Goldfinch	7.5	27	6.0	15	2.9	4
	House sparrow	4.9	26	7.8	33	3.0	10
	Linnet	16.5	23	10.3	13	5.7	6
	Nightingale	12.4	15	20.7	12	2.0	2
	Tree sparrow	10.9	11	9.3	9	0.7	1
	Barn swallow	2.7	3	0.0	0	0.4	1
	Total	17.6	774	15.4	468	4.4	142
United Kingdom							
	Blue tit	31.8	169	26.8	86	9.7	7
	Great tit	41.7	153	28.5	68	10.9	13
	Robin	36.6	108	22.3	37	4.9	5
	Blackbird	18.1	69	19.5	47	6.8	3
	Common redstart	45.6	37	21.4	9	0	0
	Chaffinch	10.6	15	9.4	8	0	0
	Long‐tailed tit	27.6	12	26.5	7	0	0
	Mistle thrush	16.2	7	12.8	4	0	0
	Song thrush	22.7	7	4.2	2	0	0
	Chiffchaff	6.8	4	8.4	3	0	0
	Blackcap	0.0	0	5.9	2	0	0
	Total	28.3	581	21.5	273	6.3	28

^a^ Only dominant species shown.

^b^ Spain: 2011 and 2012, UK: 2012, 2013, and 2014.

^c^ Spain: 2010 to 2011 and 2011 to 2012, UK: 2012 to 2013 and 2013 to 2014.

^d^ Spain: from 2011 to 2012, UK: from 2012 to 2013 and 2013 to 2014.

^e^ Mean over years, if data from more than 1 yr.

### Seasonality and year‐to‐year comparison in abundance of common bird species

The estimated population size of all investigated dominant bird species did not differ significantly between the study years (Tables 5 and 6), indicating that there was no long‐term decline in bird numbers due to the repeated application of chlorpyrifos. Because we assumed the populations to be closed, the estimated population size is constant during a whole trapping season, and within‐season variability was modeled through the detectability φ.

**Table 5:** Effect estimates from N‐mixture models of year, date of capture and exposure to chlorpyrifos on the estimated population size and detectability of birds (no. individuals trapped) in citrus orchards between 2010 and 2012^a^

	Population size *N*				Detectability φ			
	Estimate	SE	Z	*p*	Estimate	SE	Z	*p*
Blackbird								
Intercept	3.902	0.161	24.19	<0.001^*^	–3.684	0.328	–11.22	<0.001^*^
Year	0.154	0.114	1.34	0.179				
Exposure					0.131	0.335	0.390	0.697
Day of year					–0.655	0.342	–1.917	0.055
Day of year^2^					–0.304	0.116	–2.627	0.009^*^
Day of year^3^					–0.058	0.035	–1.663	0.096
Exposure × day of year					–0.292	0.364	–0.801	0.423
Great tit								
Intercept	3.889	0.242	16.10	<0.001^*^	–3.116	0.505	–6.17	<0.001^*^
Year	–0.173	0.165	–1.05	0.293				
Exposure					–1.646	0.504	–3.26	0.001^*^
Day of year					0.687	0.523	1.31	0.189
Day of year^2^					0.388	0.149	2.60	0.009^*^
Exposure × day of year					–1.938	0.598	–3.24	0.001^*^
Greenfinch								
Intercept	3.682	0.268	13.73	<0.001^*^	–3.466	1.036	–3.344	0.001^*^
Year	–0.065	0.203	–0.318	0.751	–0.373	0.620	–0.602	0.547
Exposure					–0.035	1.020	–0.034	0.973
Day of year					0.582	0.743	0.783	0.434
Day of year^2^					–0.219	0.170	–1.288	0.198
Day of year^3^					–0.058	0.054	–1.074	0.283
Exposure × day of year					–1.436	0.878	–1.636	0.102
Day of year × year					–0.648	0.402	–1.614	0.107
Day of year × exposure × year					1.471	0.449	3.279	0.001^*^
House sparrow								
Intercept	3.738	0.341	10.97	<0.001^*^	–4.615	0.823	–5.61	<0.001^*^
Year	–0.032	0.259	–0.124	0.901	1.632	0.503	3.25	0.001^*^
Exposure					0.856	0.803	1.07	0.286
Day of year					–2.034	0.697	–2.92	0.004^*^
Day of year^2^					–0.877	0.226	–3.89	<0.001^*^
Day of year^3^					–0.289	0.083	–3.49	<0.001^*^
Exposure × day of year					1.410	0.869	1.62	0.105
Day of year × year					1.583	0.335	4.73	<0.001^*^
Exposure × year					–1.440	0.488	–2.95	0.003^*^
Day of year × exposure × year					–1.616	0.417	–3.87	<0.001^*^
Sardinian Warbler								
Intercept	3.084	0.415	7.42	<0.001^*^	–3.403	0.572	–5.953	<0.001^*^
Year	0.383	0.264	1.45	0.147	0.088	0.199	0.443	0.658
Exposure					–0.151	0.456	–0.332	0.740
Day of year					–1.158	0.473	–2.447	0.014^*^
Day of year^2^					–0.254	0.210	–1.212	0.226
Day of year^3^					–0.084	0.053	–1.593	0.111
Exposure × day of year					–0.409	0.551	–0.741	0.459
Day of year × year					0.596	0.112	5.323	<0.001^*^
Serin								
Intercept	4.063	0.207	19.61	<0.001^*^	–2.773	0.609	–4.553	<0.001^*^
Year	0.114	0.154	0.737	0.461	–0.523	0.387	–1.352	0.176
Exposure					–0.412	0.598	–0.689	0.491
Day of year					–0.005	0.445	–0.012	0.991
Day of year^2^					–0.199	0.130	–1.524	0.127
Day of year^3^					–0.050	0.041	–1.244	0.214
Exposure × day of year					–1.131	0.556	–2.033	0.042^*^
Day of year × year					–0.259	0.240	–1.078	0.281
Exposure × year					0.444	0.372	1.193	0.233
Day of year × exposure × year					0.722	0.286	2.526	0.015^*^

^a^ For exposure the reference level is “not exposed”. Parameter estimates are given on the scale of the natural logarithm for the population size and on the logit scale for the detectability.

^*^ Significance levels less than 0.05.

**Table 6:** Effect estimates from N‐mixture models of year, date of capture and exposure to chlorpyrifos on the estimated population size and detectability of birds (no. individuals trapped) in cider orchards between 2012 and 2014^a^

	Population size *N*				Detectability φ			
	Estimate	SE	Z	*p*	Estimate	SE	Z	*p*
Blackbird								
Intercept	3.707	0.267	13.88	<0.001^*^	–2.856	0.211	–13.558	<0.001^*^
Year	0.141	0.155	0.91	0.363				
Exposure					0.098	0.130	0.749	0.454
Day of year					0.712	0.156	4.552	<0.001^*^
Day of year^2^					–0.464	0.073	–6.392	<0.001^*^
Day of year^3^					–0.191	0.084	–2.268	0.023^*^
Exposure × day of year					0.539	0.213	2.529	0.011^*^
Great tit								
Intercept	3.610	0.218	16.557	<0.001^*^	–2.507	0.151	–16.60	<0.001^*^
Year	0.023	0.151	0.152	0.880				
Exposure					0.003	0.137	0.025	0.980
Day of year					–0.819	0.062	–13.26	<0.001^*^
Day of year^2^					–0.141	0.058	–2.435	0.015^*^
Exposure × day of year					0.490	0.152	3.217	0.001^*^
Blue tit								
Intercept	4.061	0.195	20.80	<0.001	–2.737	0.173	–15.85	<0.001^*^
Year	0.113	0.154	0.733	0.463	–0.067	0.133	–0.503	0.615
Exposure					–0.119	0.185	–0.639	0.523
Day of year					–0.560	0.124	–4.521	<0.001^*^
Day of year^2^					–0.360	0.080	–4.533	<0.001^*^
Day of year^3^					–0.226	0.064	–3.547	<0.001^*^
Exposure × day of year					1.172	0.268	4.370	<0.001^*^
Day of year × year					–0.096	0.066	–1.460	0.144
Exposure × year					0.293	0.137	2.142	0.032^*^
Day of year × exposure × year					–0.346	0.166	–2.088	0.037^*^
Robin								
Intercept	3.472	1.44	2.408	0.016^*^	–2.708	1.519	–1.783	0.075
Year	0.256	0.75	0.341	0.733	0.038	0.778	0.049	0.961
Exposure					0.517	0.226	2.289	0.022^*^
Day of year					0.137	0.177	0.778	0.437
Day of year^2^					–0.358	0.072	–4.983	<0.001^*^
Day of year^3^					0.067	0.075	0.892	0.372
Exposure × day of year					0.034	0.321	0.106	0.916
Day of year × year					–0.159	0.080	–1.996	0.046^*^
Exposure × year					–0.453	0.165	–2.741	0.006^*^
Day of year × exposure × year					0.290	0.227	1.276	0.202

^a^ For exposure the reference level is “not exposed”. Parameter estimates are given on the scale of the natural logarithm for the population size and on the logit scale for the detectability.

^*^ Significance levels less than 0.05.

**Figure 3:**
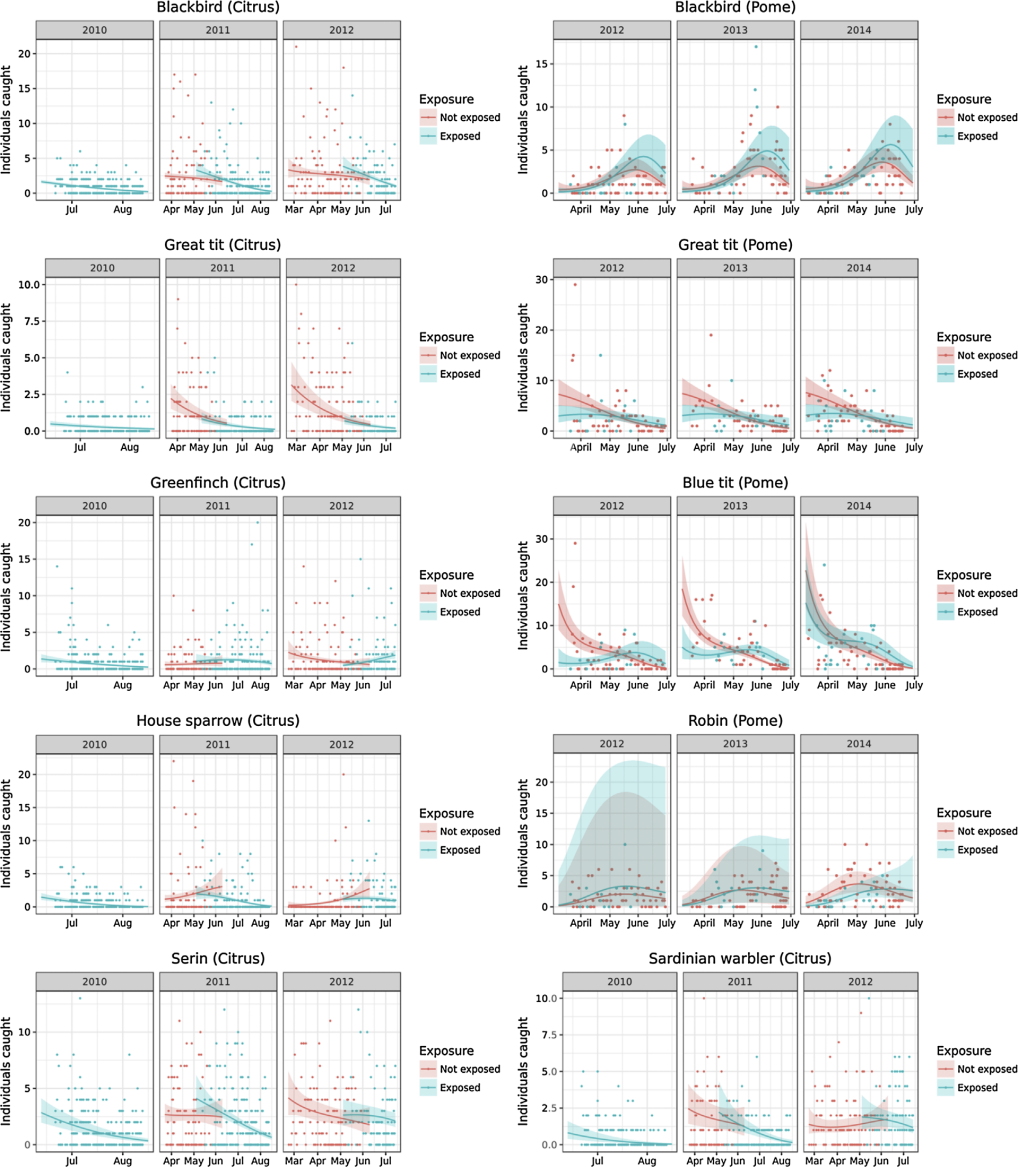
Number of adult individuals of dominant species trapped in citrus (left) and cider (right) orchards in 2010–2014. Lines indicate predictions of detectability from *N*‐mixture models with 95% confidence intervals. Note the different scales of the *y*‐axes.

At the species level, seasonality significantly affected the detectability and, hence, the number of caught birds in 4 of the 6 species (blackbird, great tit, house sparrow, Sardinian warbler) in citrus (Table 5) and all 4 common species in cider orchards (blackbird, great tit, blue tit, robin; Table 6). The effect of seasonality followed, however, different patterns among the species (Figure 3). In citrus orchards, blackbird detectability was rather constant until June and decreased afterward until the end of August, similar to serin and Sardinian warbler populations. However, the decline in detectability toward the end of the breeding season was not caused by exposure to chlorpyrifos in blackbirds and Sardinian warbler, as indicated by the *N*‐mixture model (Table 5). A decrease in the detectability of great tits (cider and citrus orchards), and blue tits (cider orchards) could be observed throughout the season. House sparrow (citrus) and robin (cider) detectability increased until the end of May and decreased afterward until the end of the observation period. The blackbird detectability in cider orchards followed a similar dynamic, but the decrease only started in June.

Exposure to chlorpyrifos was correlated with a significantly lower detectability of great tits and blue tits in cider orchards during the first half of the trapping period (Figure 3), but the numbers of exposed and not exposed birds were equal in the second half of the trapping season despite further application in cider orchards. This different detectability of exposed and not exposed great and blue tits over the trapping season is indicated by the significant interaction between exposure and day of year (Table 6). This repelling effect was less obvious in great tits in citrus orchards (Figure 3), but was indicated by the *N*‐mixture model (Table 5).

## DISCUSSION

The comprehensive long‐term monitoring program we describe here resulted in valuable insights into the bird communities in citrus and cider orchards in terms of conducting risk assessments of pesticides in general and chlorpyrifos in particular. First, it was possible to characterize the structure of the community and the presence of foraging guilds in detail. Moreover, the proportion of the population that uses a defined orchard area regularly within and between years could be calculated at the species level. Finally, temporal dynamics in the abundance and development of the population between the years were identified. This information allows us to estimate the likelihood of any long‐term effect of chlorpyrifos application over consecutive years.

### General structure of bird communities

Despite the different geographical (European) location and local differences among the study orchards, the present study shows that bird communities in commercial orchards share some common features. Twelve and 11 species in Spain and the United Kingdom, respectively, were considered abundant. Blackbird, great tit, and blackcap were present in both countries. Hence, few abundant species in the orchard communities are relevant for risk assessment. The Spanish bird community was more abundant and richer in species, whereas the United Kingdom community was more uniform and contained fewer species. The higher abundance in Spain can be explained by a general north–south gradient in bird densities (Forsman and Mönkkönen 2003), by species migrating through the country, and by the greater attractiveness of the citrus orchards as breeding and feeding habitat. Differences between countries in numbers of species might be explained as well by a greater heterogeneity of the surrounding habitat structure (Benton et al. 2003; Myczko et al. 2013).

Previous studies in orchard systems have recorded fewer bird species: 30 in French apple orchards (Bouvier et al. 2011), 26 in Italian orchards (Genghini et al. 2006), 30 in Polish apple orchards (Wiacek and Polak 2008), and 36 in Swiss apple orchards (Müller et al. 1988). The monitoring method used in those studies probably caused the difference: the constant‐effort mist netting used in the present study facilitates the detection of conspicuous and/or infrequent species, whereas other studies relied only on direct observations. In addition, the water supply in the citrus orchards due to irrigation in an otherwise dry area attracts birds into the orchards for drinking and feeding. The availability of resources on site (e.g. water, adequate nesting sites or food), and also the presence of different habitat structures in surrounding areas (Benton et al. 2003; Devictor and Jiguet 2007), are usually considered important factors in bird diversity.

### Presence of feeding guilds

In both countries, insectivorous species were the most abundant among the trapped birds. This finding underlines the importance of insectivores in risk assessment. Small insectivorous species are considered to be the most exposed to pesticide residues because of their high food intake to body weight ratio and the possibility of feeding on contaminated arthropods from treated crops.

The blackbird, representing the omnivorous guild, also proved to be a relevant species. In citrus, it was the dominant species, with the highest number of active nests found (unpublished data), probably because of the advantages of irrigated soil for feeding. Blackbirds were also commonly trapped in cider orchards, but they bred in the surrounding area. Most likely, blackbirds profit from the short vegetation under the pome trees for foraging, but the tree structure does not seem to be attractive for nesting.

Granivorous species contributed more than 33% of the trapped birds in Spain but only 9% in the United Kingdom. Four granivorous species in Spain, but only one in the United Kingdom, were among the dominant bird community. In Spain, granivorous birds established nests in the citrus trees. Despite regular mowing and herbicide applications, there were some plants with seeds available to feed on. However, these species collected most of their food outside the orchards, along paths and fallow land, as indicated by telemetry observations (unpublished data). In contrast, the cider orchards did not provide food or suitable nest possibilities for granivorous birds, which would explain the difference in the presence of granivorous species in the local orchard community and thus also the lower diversity indices of the cider orchards.

In orchards under conventional management, the use of insecticides—in general, not chlorpyrifos specifically—has been proved to affect the structure of the arthropod community, which consequently reduces the abundance and diversity of the bird community (Genghini et al. 2006; Simon et al. 2010). Indirect effects of food depletion by insecticide application may also reduce the breeding performance of insectivorous birds (e.g., Boatman et al. 2004), and it has been shown that insectivorous bird species profit from organic farming practices (Bouvier et al. 2005; Genghini et al. 2006). The supply of insects, whose density is probably increased on organic farms where chemical treatments are less intense, is likely to be the main reason (Dritschilo and Wanner 1980; Moreby et al. 1994; Weibull et al. 2003). Therefore, it was surprising to find that regardless of regular applications of a broad‐band insecticide like chlorpyrifos over many years, insectivorous species were the most abundant feeding guild in both orchard systems. After installation of nest boxes in pome trees without natural cavities, a population of 2 insectivorous tit species was successfully established inside the cider orchards (unpublished data), underlining the fact that for breeding activities, the lack of nesting sites had a higher importance than the application of chlorpyrifos.

### Site fidelity

To study the potential effects of exposure to plant protection products (PPPs), individuals closely associated with the study orchards for breeding and/or foraging are of high interest. They can be identified as the proportion of the population that was trapped again within the same study period and/or returned in subsequent years to the same area.

There were considerable differences in these proportions (within‐ or between‐season retraps) by species, presumably due to differences in territory size and general use of the orchard. Breeding inside the orchards particularly increased retrapping rates: blackbirds bred inside citrus orchards, whereas in the United Kingdom, they used the orchards only later in the season, and for feeding. In contrast, the serin was an abundant breeding species with low retrapping rates. Telemetry data showed that their home range was significantly larger than the study orchards, which decreases the time an individual spends in a certain area and, accordingly, the chance of retrapping but also of exposure. Robins were more likely to be retrapped than blackbirds; even though robins use the orchards for foraging alone, they have smaller home ranges than blackbirds (unpublished data). Hence, to verify potential effects of PPP application, the focus should be on the species that use the orchard most regularly. In the present study, these were the great tit in both countries, the blackbird and Sardinian warbler in Spain, and the blue tit and robin in the United Kingdom.

Breeding site fidelity in adult birds is known to be more pronounced than natal site fidelity in juveniles (e.g., Weatherhead and Forbes 1994; Divoky and Horton 1995; Vadász et al. 2008). Therefore, site fidelity between years can be used to assess habitat quality for birds (e.g., Ingold et al. 2010). Individuals that consider a territory to be of high quality tend to return for breeding in the following season. In general, the species percentages of between‐season retraps, as an outcome of survival and site fidelity, were lower but in a similar range as the within‐season retraps, as a measure of the local population size. Thus, a large proportion of the local population in chlorpyrifos‐treated orchards survived between seasons and perceived the orchards to be a high quality habitat.

### Seasonality and short‐term effects of chlorpyrifos application

Distinct seasonal patterns in detectability were found for some species, probably connected to the specific life cycles. In both countries, insectivorous tit species followed similar trends, and their detectability decreased throughout the season. At the end of their breeding period, adult tits usually leave the breeding area together with the fledged chicks. In citrus orchards, this decrease in detectability coincides with the chlorpyrifos application period, and therefore a connection between both factors seems plausible. As the arthropod mass is noticeably reduced after the applications (authors’ own observations), the adult birds moved into off‐crop habitats to forage (unpublished data). A similar pattern is likely to exist for great and blue tits in the cider orchards, where fewer numbers of these species were detected after chlorpyrifos applications in April and May; the tits were probably foraging outside the orchards. In general, the species’ seasonal patterns of abundance can be used for defining the focal species more precisely according to the timing of the application period.

Assessment of mortality after application periods is also an important way of estimating pesticide effects on birds. This was not a specific aim of the present study, because the acute effects have already been assessed by Wolf et al. (2010) using telemetric monitoring on a wide scale in a multiple‐crop and multiple‐country approach.

### Long‐term effect of chlorpyrifos application

In the present study, none of the species were found to decline in abundance after consecutive years of insecticide use. The population sizes were rather constant or developed positively for all species.

## CONCLUSIONS

The documented long‐term utilization of chlorpyrifos‐treated citrus orchards by bird individuals and the stable population sizes indicate the presence of sustainable bird communities within both study areas. Our detailed analysis of temporal dynamics in the bird communities and their orchard use might aid in further risk assessments of PPPs. The present study further showed that agricultural management practices (e.g., regular mowing, which decreases the supply of herb seeds, or irrigation as the water supply in a dry environment) might affect the local bird community far more than the application of a pesticide.

## Supplemental Data

The Supplemental Data are available on the Wiley Online Library at DOI: 10.1002/etc.4317.

## Conflict of Interest

The authors declare that they have no conflicts of interest. As noted, funding for all expenses was from Dow AgroSciences and ADAMA. The study design was discussed with the sponsors, but they had no role in the implementation, data collection, or interpretation of results. The study design was intended to simulate good agricultural practice.

## Supporting information

This article includes online‐only Supplemental Data.

Supporting Data S1.

## Data Availability

Research data pertaining to the present study are located at https://figshare.com.
