# Supplementary material for: Multiyear monitoring of bird communities in chlorpyrifos‐treated orchards in Spain and the United Kingdom: Spatial and temporal trends in species composition, abundance, and site fidelity
Source: Environ Toxicol Chem. 2019 Jan 23;38(3):616–29. doi: 10.1002/etc.4317 (PMC6850510; doi:10.1002/etc.4317)
Supplement: Supplementary file 1 — Supporting Data S1. [file ETC-38-616-s001.pdf]

Supplemental Data

Multi-year monitoring of bird communities in chlorpyrifos-treated orchards in Spain and UK: Spatial and temporal trends in species-composition, abundance and site-fidelity

by

Ralf Dittrich, Benedikt Giessing, María M. Benito, Anja Ruß, Christian Wolf, Manousos Foudoulakis, Steve Norman

Table S 1: Characteristics of study sites in Spain

Site	Area [ha]	Centre point of study site (WGS 84 / UTM zone 30S)		Average height of trees [m]	Maximum height of trees [m]	Cultivar	Planting Year
		Easting	Northing				
1	5.2	701844	4312909	2.2	6.0	Marisol	1991
2	6.7	712851	4314311	2.4	3.2	Lane Late, Clemenvilla	2000
3	5.3	705335	4312384	2.3	3.0	Lane Late, Clemenvilla	1991
4	3.7	708265	4315389	2.4	3.0	Navelina	1985
A4	2.6	708117	4315306	2.4	3.0	Navelina, Clemenvilla	n.a.
5	4.5	713114	4320256	2.6	4.0	Navelina, Clemenules	1986
6	4.3	702891	4310530	2.4	3.2	Orogrande	1990
7	4.4	721083	4321992	2.3	2.5	Okitsu	1996
8	3.5	706126	4314038	2.8	3.5	Navelina	2000
9	4.4	701278	4309520	3.1	4.0	Navelina	2001
10	8.4	699104	4309764	2.3	2.5	Arrufatina, Clement Rubí, Oronules, Navel Powel summer	2002

Table S 2: Characteristics of study sites in UK

Site	Area [ha]	Centre point of study site (WGS 84 / UTM zone 30N)		Attribution
		Easting	Northing	
1	4	533199	5765059	Conventional
2	5	524162	5764742	Conventional
3	6	533598	5765723	Conventional
4	9	534790	5773999	Conventional
5	10	515377	5767625	Conventional
6	5	514493	5767860	Conventional
7	8	508718	5780235	Conventional
8	8	506121	5771279	Conventional
9	6	504622	5770949	Conventional
10	7	504984	5772939	Conventional
11	9	516072	5766733	Reference
12	3	524189	5762025	Reference
13	3	533166	5760545	Reference

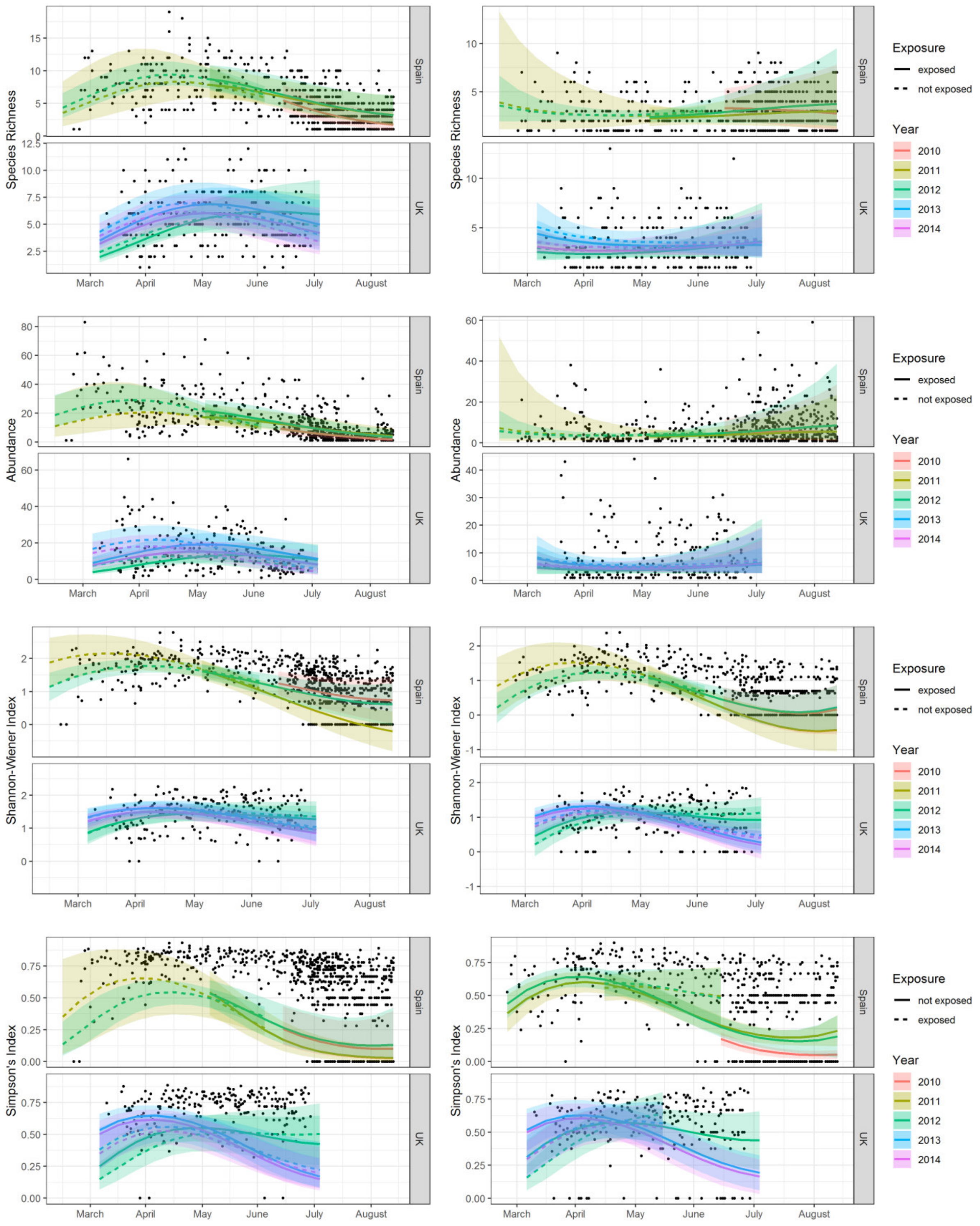

Figure S 1 Session-wise community indices for trapped birds in citrus (2010-2012) and cider orchards (2012-2014). The left column shows indices and predictions from analysis including all trapped birds, the right column includes only insectivorous species. Lines indicate predictions from GLMMs with 95% confidence intervals.
